# Nasal Obstructions Which Result in Mouth-Breathing, with Special Reference to Adenoid Vegetations

**Published:** 1896-09

**Authors:** George L. Richards

**Affiliations:** M.D. (Harv.), Boston, Mass.


### International Dental Journal.

VOL. XVII. SEPTEMBER, 1896. No. 9.

### Original Communications.[1]

### NASAL OBSTRUCTIONS WHICH RESULT IN MOUTH-BREATHING, WITH SPECIAL REFERENCE TO ADENOID VEGETATIONS.[2]

BY GEORGE L. RICHARDS, M.D. (HARV.), BOSTON, MASS.[3]

BEFORE taking up the main subject which I wish to consider this evening, it may be well for us briefly to review our knowledge of the anatomy and physiology of the nasal organs, as only by a proper appreciation of their normal anatomy and physiology can we hope to understand their pathology and treatment.

Anatomically, the nose is the beginning of the upper air-tract, being continued below by the pharynx, larynx, and trachea. In construction it is both bony and cartilaginous, and consists essentially of two cavities—a right and a left—separated by a partition wall,—the septum. The septum is cartilaginous in front, bony behind. The bony portion is formed by the *vomer*, beginning at the posterior superior border of the choanæ and extending in a gradually descending line forward almost to the anterior nasal opening, and the perpendicular plate of the ethmoid, which overhangs the vomer and extends to the lower border of the nasal bone. The anterior inferior portion is called the lamina quadrangularis, or quadrilateral cartilage, and remains for the greater part of

[1] The editor and publishers are not responsible for the views of authors of papers published in this department, nor for any claim to novelty, or otherwise, that may be made by them. No papers will be received for this department that have appeared in any other journal published in the country.

[2] Read before the Harvard Odontological Society, November 21, 1895.

[3] Matriculate of the Universities of Vienna and Halle.

life cartilaginous. It is important to remember that the septum reaches above to the lamina cribrosa of the ethmoid, and that through this pass the filaments of the olfactory nerve, to be distributed over the upper two-thirds of the septum. Projecting into the cavity of each nostril are the inferior, middle, and superior turbinate bones. The inferior turbinate is a separate bone, and lies on the superior maxilla, partially covering the opening into the antrum, and extending a little behind the posterior choana. The superior and middle turbinates are a part of the ethmoid bone, and are attached to the vertical plate. The superior is visible from behind only, while the middle and inferior are visible from in front. All these bones have a concave and a convex surface, the convex surface presenting towards the septum. Opening into the nose are the outlets of a number of accessory cavities,—viz., the antrum, frontal sinus, anterior ethmoidal cells, posterior ethmoidal cells, and sphenoidal cells. These cavities are already indicated in the new-born, and reach their full size soon after the second dentition.

The antrum, the largest of the accessory cavities, lies in the superior maxilla, its opening, the hiatus maxillaris, being partly closed by the laying over it of the inferior turbinate, together with the processus uncinatus of the ethmoid. The spaces remaining in the bony wall are covered with mucous membrane, with the exception of the normal opening into the hiatus semilunaris. The antrum comes in front as far forward as the inferior turbinate, behind not so far; above it reaches to the orbit, below to the alveolar processes of the teeth (viz., the last bicuspid and first two molars), —*i.e.*, lower than the floor of the nose.

The frontal sinus begins at the root of the nose, occupies the region over the eyebrows, and reaches half as high as the forehead. It extends outward about two inches (five centimetres). The partition wall is almost never in the middle line, but more to one side or the other. The two never communicate. Below, the sinus extends usually to the region of the anterior end of the middle turbinate, and its opening will be found in the hiatus directly in front of that of the antrum.

The ethmoid cells lie over the middle meatus. They have no direct outlets except orifices, with simple gaps in the outer and upper walls of the middle meatus. The anterior and posterior cells open often in the superior meatus also. One of the anterior ethmoidal cells is especially prominent, and forms the outer wall of the hiatus semilunaris. This is the bulla ethmoidalis,—somewhat larger than the other ethmoid cells, and projecting forward as a spherical mass. Not seldom is an ethmoid cell found in the anterior portion of the middle turbinate bone. When considerably enlarged it forms the so-called bony enlargement of the anterior end of the middle turbinate. This can reach the size of a nut. On the outer side the ethmoid cells reach to the orbital cavity; consequently it can come to pass that in affections of these cells the eye can be pushed outward and downward, causing double sight.

The sphenoid sinus lies in the body of that bone behind the posterior wall of the nose, above the choanæ. Its walls are thin in adults, with the exception of the inferior, which separates it from the naso-pharynx. This is usually thicker. The cavity is always somewhat higher than the middle turbinate of that side. With the head in the usual posture for a nasal examination, a straight probe passed from the floor of the nose at the entrance to the middle portion of the middle turbinate, and then onward, would strike exactly the sphenoid cavity. The natural opening lies somewhat higher than the posterior end of the middle turbinate, and behind the superior meatus.

The naso-pharyngeal cavity is bounded in front by the choanæ; on the side by the posterior nasal sulcus, the tube-opening, the cartilage of the Eustachian tube, and the fossa of Rosenmüller; posteriorly, by the axis, atlas, and occipital bones; the roof is formed by the occipital and sphenoid. The velum palati is the anterior inferior border. The anterior lip of the Eustachian tube is much the smaller; the posterior lip is extended downward to the salpingo-pharyngeal fold. The fossa of Rosenmüller lies behind and above the tube-opening.

The tonsils lie between the two palatine arches, and may normally project a little beyond the anterior arch. They contain many lacunæ, which act as sources of trouble in affections of this organ. The relation of the tonsil to the great vessels in the neck is important. They lie quite a distance behind and to the outer side of the tonsil; hence there is little danger of their injury in the operation of tonsillotomy. Bleeding after tonsillotomy comes either from parenchymatous bleeding, due to abnormal widening *of* or changes *in* the twigs of the tonsillar artery, or from cutting a twig of an artery of some size. The tonsillar artery is not constant in origin, sometimes coming from the ascending palatine or external maxillary or ascending pharyngeal, even direct from the carotid. The external maxillary can come very close to the tonsil, and the ascending pharyngeal as well.

The nose, with its accessory cavities, is lined with mucous membrane, which, except where there is spongy tissue, is very closely attached to the periosteum or perichondrium. In the upper portion of the nose, that concerned more especially with the sense of smell, the epithelium is ciliated, while in the lower, more concerned in breathing, the epithelium is pavement celled, the border line, which is not constant, lying in the middle meatus. Mucous glands are the only ones present, and they are abundant. Cavernous tissue covers the inferior turbinate and a portion of the anterior end of the middle turbinate. It consists of connective tissue, stroma, smooth muscle-fibres, and elastic tissue. Underneath the subepithelial layer is a net-work of sinuous, wide, anastomosing veins that course at right angles to the surface of the turbinate tissue. In order to provide blood the finer arteries course corkscrew-like through the tissues. This tissue can swell so as to touch the septum, or contract so as to almost disappear. It is presided over by the sympathetic branches of the trigeminus, coming from the spheno-palatine ganglion. The blood-supply of the nose is very abundant, coming mostly from branches of the internal maxillary. The veins communicate with those of the brain.

The olfactory nerve, passing through the cribriform plate, spreads itself out on both sides of the septum, down as far as the middle meatus. Consequently smell is limited to this region.

Physiologically, the nose has three important functions: First, as the seat of the sense of smell. Second, it assists in the formation of the voice, producing the overtones, and, in connection with the naso-pharynx, acting as a resonator. In the production of most sounds the soft palate is raised against the pharyngeal wall and the nasal cavity is more or less cut off, while in uttering *m*, *n*, or *ng*, the purely nasal tones, the palate is relaxed, and the tone passes through the nose with the expired air. For the production of a good voice it is absolutely essential that the whole upper air-tract be free from obstruction. Third, it is the beginning of the respiratory tract, and, as a receiver of the outside air and carriers of the same to the pharynx on its way to the trachea and lungs, warms and moistens the inspired air, and in a measure removes the dust.

As a result of the experiments of Bloch, Aschenbrandt, and Kayser, the following important facts have been demonstrated:

*First.* The temperature of the inspired air after passing through the nose is considerably elevated, according to the following formula, in which $E =$ the degree of warming, $v =$ body temperature, $t =$ temperature of the outside air (Bloch): $E = \frac{5}{9} v - t$. If the temperature of the outside air is 40°, then $E = \frac{5}{9}$ (98.6 — 40); $\frac{5}{9}$ (58.6)

= 32.6, which added to 40 = 72.6, the temperature after leaving the nose. Other authorities say the air is raised always to 86°. The relatively narrow canal, but really great area of surface over which the air passes in the nose, satisfactorily explains this. The amount of warming is considerably less when air is breathed through the mouth. Expired air is one to three degrees warmer than inspired.

*Second.* Inspired air on leaving the nose is nearly saturated with moisture, receiving from five to seven thousand grains in twenty-four hours.

The blood-supply of the cavernous tissue, from whence the heat and moisture mostly come, has a direct relation to the degree of cold and moisture of the external air, being increased as cold and dryness are present, and *vice versa.* In mouth-breathing the amount of moisture absorbed is less; furthermore, the mouth feels dry as a result, as any one who has awakened in the morning after a night of mouth-breathing well knows.

While the nose is not a perfect dust-collector, still it retains the greater part of the dust inhaled, which either remains there or is blown out by the current of expired air. In the mouth there is no provision for dust-collection.

It becomes, then, evident that the nose in its normal condition is perfectly adapted for the breathing function, no matter what the variations of temperature or moisture, and that the mouth cannot normally take its place or properly do its work. Whence it follows that mouth-breathing must be always pathological, and dependent upon nasal or naso-pharyngeal obstruction in some form.

Among the pathological symptoms which accompany mouth-breathing are: Bronchitis of varying degree, due to the inhalation of unclean, too dry, and insufficiently warmed air; tendency to the infectious troubles of the air-tract. Pharyngitis and laryngitis, both follicular and dry, of varying degree, with symptoms of cough, sore throat, hoarseness, and dyspnœa. Alterations in the voice, due to interference with formation of the nasal tones; voice easily tired; stammering. Dry mouth. Coated tongue and other digestive disturbances. Open mouth. Noisy respiration in the daytime, and snoring at night. A peculiar facial expression, alæ nasi flattened, pinched, or dimpled, bridge of nose often widened, and the veins thereabout dilated. Abnormalities of secretion, with inflammation of nasal mucous membrane, crust formation, fissures and cracks at junction of nose and lips, thickened lips, excessive lachrymation and nose-bleeding. Herpes and eczema may complicate. Chest deformities. Deafness. Loss of sense of smell. Dulness of intellect, headache, backwardness, derangements of sleep, temper, spirits, energy, melancholia. Sneezing and reflex neuroses, including asthma, epilepsy, chorea, convulsions, stammering, aphonia, pertussis.

It may be thought that I have drawn a much exaggerated picture of the pathological possibilities, but such, I think, is not the case. Among the common subjective evidences of nasal stenosis, with accompanying mouth-breathing, as evidenced by the complaints of the patient, are the following: Sense of fulness in nose and head, a sensation as though there was a foreign body present, as in case of polypi. Dryness in mouth, pharynx, and larynx, especially on waking in the morning. Headache and frontal pain. Inability to breathe through the nose; this partial only or complete, as the case may be. Sore throat and altered voice. Impairment of smell and taste. Asthma. Chilliness or feverishness. Deafness and tinnitus. Disorders of common sensations.

The *causes* of nasal obstruction, arranged somewhat in the order of their frequency, are: Adenoid vegetations in the naso-pharynx. Hypertrophied tonsils, especially those which tend to meet in the middle line, and, with the arch of the tongue, block up the pharynx when the mouth is closed. Injuries to the nose from external violence having as their result septal deviations and spurs. These injuries have often taken place in early childhood, accompanied by a nose-bleed, and later have been forgotten. Hypertrophies of the turbinate tissue. Polypi. Foreign bodies. Syphilis, secondary, tertiary, and acquired. Malignant growths. Congenital atresia. Tuberculosis. These latter causes are beyond the scope of the present paper, as I wish here to consider only the more common causes of nasal obstruction, especially adenoids, particularly as seen in children.

In the naso-pharynx of young persons swellings are frequently found due to hyperplasia of the adenoid tissue normally there present. Meyer, of Copenhagen, was the first to call attention to the significance and importance of these growths. They appear in two principal forms. The first as a tumor-like growth, much resembling the normal tonsil, situated in the posterior superior wall of the naso-pharynx, often encroaching upon the point of origin of the Eustachian tube. This form has numerous furrows, the deepest being in the middle. The second form consists of a central mass or masses, with numerous projections hanging downward. With this form the whole mucous membrane of the naso-pharynx may be involved, the projections being inverted cones, but all more or less connected at their bases. There is also great variation in the absolute amount of the growth, varying from a few projections to complete filling of the naso-pharyngeal vault down to the floor of the choanæ. The first form is the more common, and is a much more solid growth than the second. Both forms are abundantly supplied with blood-vessels, very poorly supplied with nerves. They bleed readily, especially the second form. In consistence the first form is fairly firm; the second very soft, and feeling, one writer has said, much like a bag of worms under the finger. In structure they consist of a connective-tissue framework, filled with lymph-cells (nucleated), mucous glands, and many blood-vessels. The epithelium covering them is of the ciliated, cylinder type. They may show their presence in the new-born, but are most common from five to fifteen years of age, more seldom from fifteen to twenty, still more seldom before five or after twenty. After the age of puberty they not infrequently undergo spontaneous involution, the same as the normal adenoid tissue. Both sexes are affected in about the same ratio.

The cause is not very clear. Heredity and climate, especially the latter, seem to play a part, as they are found more frequently in the north and on the coast than inland and to the south. Scrofula, measles, scarlet fever, and whooping cough are also causative. In many cases obstruction in the anterior part of the nose has seemed to favor the growth. In still other cases no satisfactory cause has been found.

They produce trouble on account of their mechanical hinderance to the proper performance of the function of the naso-pharynx, and by reason also of the disorders in the secretions which they bring about in this region. By even moderate development they interfere very decidedly with breathing through the nose. The clinical picture is sufficiently common to be recognized readily by us all, provided the symptoms which the growth produces are borne in mind. The disturbance is most marked in childhood. The child snores at night, and breathes through the mouth in the daytime; cannot blow the nose properly; snuffs and snorts; the voice has a harsh character from the resultant laryngeal irritation. The nose has a flattened appearance; at the tip the alæ are widened. The face has a more or less vacant look. The nasal sounds are muffled,—*m*, *n*, and *ng* very much so. A good test are the words *music* and *many*,—"many men of many minds." The nose is apt to be full of slime, which cannot wholly be blown out; thick secretion is present in the pharynx or found hanging down from the naso-pharynx. In about one-fifth of the cases the tonsils are enlarged, and granular pharyngitis is often present. Zarniko says that a high palate and anomalies of the teeth are found after the seventh year. Affections of the middle ear are present in three-fourths of the cases, especially catarrh of the tubes and purulent inflammation of the middle ear. Killian found in five hundred and forty seven cases of ear disease one hundred and one with adenoid growths, or 18.46 per cent. In ear affections of young children the naso-pharynx should always be examined. By anterior rhinoscopy, if the passages are roomy, the vegetations may be seen from the front, covered with some thick slime. Posterior rhinoscopy (very often not possible) will show a mass of adenoid tissue projecting from the roof of the naso-pharynx, thickest at the centre, hanging down over the choanæ and obstructing the free entrance of air. Sideways they encroach upon and even overhang the cartilages of the Eustachian tube, interfering with the valve-like action of the mouth-tubes, and preventing the easy inflow and outflow of air.

The growths vary in consistence from early to late childhood, being softer the younger the child. As the child grows the growths become less sessile, more compact, and finally, at about the age of puberty, sensibly diminish. They may, however, remain, and be an obstruction in adult life. In any event, the deformities and habits consequent upon years of mouth-breathing do not disappear with the shrinkage of the growth, while the high-arched palate, contracted nasal chambers, small round nostrils, and listless facial expression remain. Furthermore, there is frequently by this time a degree of impaired hearing which no future treatment can wholly remedy. In young children, where posterior rhinoscopy is usually impossible, the forefinger, guarded with a shield of rubber, or even by a piece of towel, can be passed up behind the soft palate just above the tonsil. It is then swept over the vault, and the presence or absence of the growths noted. When, as is often the case in quite young children, the growths are soft and not abundant, they can be removed with the finger-nail at the moment of examination. The amount of pain caused is not very great. The child is bound to cry, in any event, and some slight bleeding always follows the examination. Where the growths are abundant and the degree of obstruction considerable, ether to complete anæsthesia should always be given, and the operation done with care and without hurry. I am inclined to regard the finger-nail operation as poor surgery, and very apt to be incomplete, owing to the liability of leaving some of the growth behind. (This may return to a certain degree.)

In adults of fair will-power cocaine anæsthesia suffices. As a rule, in Germany no anæsthetic other than cocaine is used for either children or adults. The choice of an instrument and the particular mode of operating depends on the preferences of the individual operator. Some form of cutting forceps is used by many, but my own preference is for the Gottstein sharp curette or knife. It is a very safe instrument, and, if properly curved and properly held, cannot fail to remove all the growth. Three introductions are usually all that is necessary: first in the middle line, and then on each side, carrying it always to the top of the vault and close to the septum before beginning, then carrying it backward over the whole surface of the vault, bearing in mind the normal curvature. If the instrument is improperly held, it scarifies the mucous membrane only, and does not remove all the growth. The resultant hemorrhage is profuse for a few minutes, but soon ceases. Sweeping the finger-nail over the denuded surface after the operation gives us information as to the completeness of removal and lessens the hemorrhage. The operation may be done in either the upright or the horizontal position. When done in the upright position the child is firmly held by an assistant, facing a good light, the operator sitting in front. The mouth being held open with a good mouth-gag and the tongue held down with a tongue-depressor, the Gottstein curette is introduced behind the soft palate, to the vault, brought forward until it touches the septum, and then swept over the vault backward and downward. The instrument is withdrawn, and at the same moment the child's head is drawn quickly forward and downward, so that the blood and the piece removed drop into a dish at the side of the assistant. As soon as the bleeding subsides a little, the head is raised, the naso-pharynx swabbed with cotton or gauze, and the Gottstein curette again introduced, proceeding in this manner until all the growths are removed. As soon as the vault is clean the child is held head downward until the bleeding ceases.

For fear that blood or portions of the removed growth may enter the larynx or trachea, some operators place the child in the recumbent posture with the head over the edge of a table. The nose and mouth being now at the most dependent point, there is no danger of the entrance of blood into the larynx. I have tried this method, but prefer the operation in the upright position, and regard this when done with the aid of competent assistance as practically devoid of danger. Complete healing of the denuded mucous membrane takes about four weeks, though the soreness immediately following lasts but a few days. A light diet and confinement to the house for a few days constitute the after-treatment. I usually also order an alkaline antiseptic spray for the nose and throat, to be used for a week or ten days. There is, of course, a slight risk of infection from the denuded surface, but practically there are no ill results. There may be a slight rise of temperature for the first twenty-four hours.

The operation *always benefits*, and should be urged upon parents in all cases where it is evident that the obstruction is considerable and dependent upon adenoids. While the good effect as regards freer breathing and improved speech comes at once, the improvement in the condition of the whole organism is best observed several months afterwards.

Time will not permit me to go into the details of the treatment of the other causes of nasal obstruction. Suffice it to say that turbinal hypertrophies should be reduced, spurs removed, septal deviations straightened, polypi and foreign bodies removed, syphilitic affections treated, and malignant growths handled according to the indications in each particular case.

I would also urge that recent injuries of the nose be carefully examined for any cartilaginous bending or breaking, which, easily rectified at the time, will later result in septal deviation or spur-formation that may result in serious occlusion of the lumen of one or both nasal passages. This is especially important in children, where beyond taking note of the nose-bleed at the time it occurs no notice is taken of such injuries.

Hypertrophied tonsils very frequently go hand in hand with adenoid vegetations, and when so present should be removed. They also exist independently. When large they frequently cause mouth-breathing, not only filling up the anterior and posterior pillars, but extending towards each other in such a way as to seriously encroach upon the pharyngeal space when the mouth is open. When the mouth is closed, and the tongue pushes the tonsils and uvula backward, the pharyngeal space may be almost completely occluded, and the free passage of air from larynx to naso-pharynx cut off. If at the same time adenoids are present, nasal obstruction may be complete. Removal of hypertrophied tonsils is usually easily accomplished with the Mathieu or Mackenzie instrument; they are sometimes so very flat and thick, and project so slightly beyond the anterior pillars as to render sufficient removal by these means impossible. In that case Leland's tonsil-knives, followed by the galvano-cautery, can be used to still further reduce them.

Tonsillotomy is easily done without ether, and the danger of bleeding is usually not great.

In conclusion, I would urge that the practitioner never order anything for the nose or for so-called nasal catarrh, be it spray, powder, or internal medicine, without first thoroughly examining the nose under good illumination, and with a good speculum. Were this more thoroughly done by the general practitioner, many a case that now goes to the specialist could be treated by the family physician.

