# Correction of Dr. Richards’s Paper

**Published:** 1897-01

**Authors:** 


### CORRECTION OF DR. RICHARDS'S PAPER.

In Dr. Richards's paper on "Nasal Obstructions," September issue, on—

Page 550, line 11 from bottom, instead of "never communicate" read "occasionally though very rarely communicate."

Page 552, line 4, instead of "ciliated" read "cylindrical."

Page 552, line 5, instead of pavement-celled" read "ciliated."

